# Supplementary material for: Conditional chemoconnectomics (cCCTomics) as a strategy for efficient and conditional targeting of chemical transmission
Source: eLife. 2024 Apr 30;12:RP91927. doi: 10.7554/eLife.91927 (PMC11060718; doi:10.7554/eLife.91927)
Supplement: Figure 3—source data 3. [file elife-91927-fig3-data3.zip › Figure 3 source data3 part 1.zip!/Figure 3-Source data 3-part1/1081-1-CRISPResso_Report_8zosk6yj.zip!/CRISPResso_on_1081_2/3a.Indel_size_distribution.pdf]

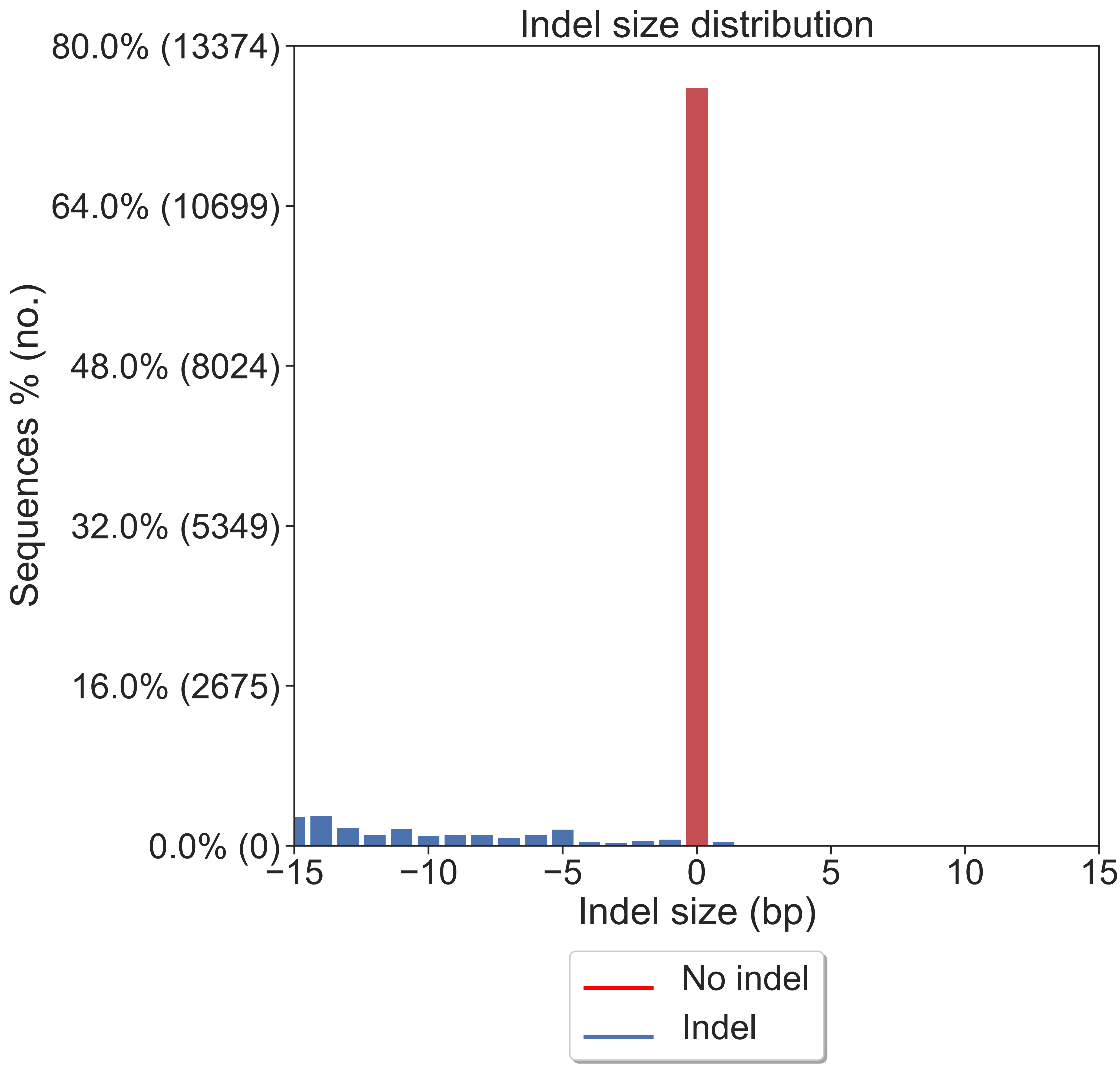

Indel size distribution
80.0% (13374)
64.0% (10699)
48.0% (8024)
32.0% (5349)
16.0% (2675)
0.0% (0)
Sequences % (no.)
−15
−10
−5
0
5
10
15
Indel size (bp)
No indel
Indel
